# Supplementary material for: Parameter tuning differentiates granule cell subtypes enriching transmission properties at the cerebellum input stage
Source: Commun Biol. 2020 May 8;3:222. doi: 10.1038/s42003-020-0953-x (PMC7210112; doi:10.1038/s42003-020-0953-x)
Supplement: Supplementary file 1 — Supplementary Information [file 42003_2020_953_MOESM1_ESM.pdf]

SUPPLEMENTARY NOTES

List of abbreviations used below:

- GrC, granule cell
- AIS, Axon Initial Segment;
- AA, ascending axon;
- PF, parallel fibers.

Ionic channels

Nav1.6 – The spike generation and propagation mechanisms exploited different Na^+ channels in the axonal hillock and initial segment (Nav1.6-FHF) with respect to AA and PF (Nav1.6) [1-6] depending of modulation by FHF (Fibroblast Grow Factor Homologous Factor). Therefore we developed two versions of the Nav1.6 sodium channel: 1) without FHF in AA and PF, and 2) with FHF in Hillock and AIS [6]. The channel without FHF was taken from [7]. The channel with FHF was based [4] and modified with a faster kinetic for the L_{off} transition (from 0.15/ms to 0.5/ms). The FHF channel in the AIS had a higher conductance compared to channels in the other section, in order to compensate for the absence of somatic Na^+ channels.

Kv1.1, Kv1.5, Kv2, Kv3.4, Kv4.3, Kv7/Km – To counterbalance the resurgent current of both types of Na^+ currents, the axonal sections were endowed with the Kv3.4 K^+ channel [1, 8, 9]. The A-type K^+ current (Kv4.3) was taken from the canonical mono and multi compartmental models [7, 10] and placed on the soma. To reduce the Ca^{2+} excitability [11], the K^+ channel Kv1.1 was placed, alone, on the dendrites and mixed with Kv1.5 on the soma [12]. The K^+ current Kv2, taken from Channelpedia (http://channelpedia.epfl.ch/ionchannels/193), was placed on the soma to act as slow delayed rectifying channel [13, 14]. The GrC M-current like slow current (Kv7), taken from the mono compartmental model [10] was placed on the AIS, instead of the soma, in accordance with its anchoring system which linked it to Ankyrin-G enriched zones, usually found in the AIS [15].

Cav2.2, KCa1.1 – The GrC N-type high-voltage activated Ca^{2+} channel[16, 17], Cav2.2, was taken from the previous mono compartmental model [10] and distributed along the entire morphology. The main Ca^{2+} dependent K^{+} channels, KCa1.1 was placed only on the dendrites.

Kir2.x – The GrC K^{+} inward rectifier channel [18, 19] was taken from [7, 10] and placed in the soma.

TRPM - A TRPM - like current was taken from the UBC multicompartmental model [20], modified to interact with Calmodulin [21] in the 2C conformation (Cam2C) [22] when reached a certain threshold. This channel was placed only on the dendrites.

Calcium (Ca^{2+}) buffer - GrC are known to buffer intracellular Ca^{2+} through Calretinin [23, 24]. To account for this, the Ca^{2+} buffer previously used by [9, 25] was modified by changing Calbindin and Parvalbumin with the 12 states kinetic Calretinin kinetic model [26]. The basal concentration of the protein was set accordingly to experimental data [27].

SUPPLEMENTARY TABLES

Supplementary Table 1. Electrotonic compartments in the GrC model.

Section name	*Diameter (µm)*	*Length (µm)*	*N° of sections*	*Cm* (µF/cm²)	*Leak Rp*	*Leak Gmax* (S/cm²)
**Dendrites**	0.75	15	4	2.5	-60	0.0002 - 0.0004
**Soma**	5.8	5.62	1	2	-60	0.0002 - 0.0004
**Hillock**	1.5	1	1	2	-60	0.0002 - 0.0004
**AIS**	0.7	10	1	1	-60	0.0002 - 0.0004
**AA**	0.3	126	18	1	-60	0.0002 - 0.0004
**PF**	0.15	980	140	1	-60	0. 0000001 - 0.0000008

The table shows the sections of the GrC model along with their diameter, length and number. GrC model dendrites, soma and hillock were taken unmodified from [25], whereas the morphology was extended to accommodate the AIS along with a specific mechanism for spike generation [28]. The AIS was built as a single section 10 µm long and 0.7 µm wide [5, 13]. The AA and PF were built connecting 7 µm modules until reaching the desired axonal length [29]. The present model consisted of an AA composed by 18 sections (total length of 126 µm) and two PF branches composed of 140 sections (1 mm each). The diameters were set to 0.3 µm for AA and 0.15 µm for PF [5]. Membrane capacitance (C_m) was set at 1 µF/cm^2 in the AIS, AA and PFs, 2.5 µF/cm^2 in the dendrites and 2 µF/cm^2 in the soma, hillock (these higher C_m values were used to normalize spike amplitude). The axial resistance (R_a) was set at 100 Ω*cm in the entire model. The input resistance (R_{in}), calculated from a 10 mV current transient (from -70 to -80 mV), in voltage-clamp mode, was in the range of 1.2-1.8 GΩ (e.g. [30]).

Supplementary Table 2. Ionic mechanisms in the GrC model.

Conductance/ Location		Range G_{i-max} (S/cm^2)	E_{rev} (mV)	Description of channel (HH or Markovian)	Reference
**Na^+ channels**					
Nav1.6 FHF	Hillock	0.008 - 0.05	87.39	Markovian	[3, 31]
	AIS	1 – 1.65			
Nav1.6 No FHF	AA	0.02 - 0.05			
	PF	0.01 - 0.04			
**K^+ channels**					
Kv1.1	Dendrites	0.0001 - 0.01	-88	HH	[32]
	Soma	0.003 – 0.01			
Kv1.5	Soma	0.13e-4 - 0.13e-2	-88	HH	[33]
Kv2	Soma	0.000001 - 0.0001	-88	HH	[14]
Kv3.4	Soma	0.0005 - 0.005	-88	HH	[1, 34]
	Hillock	0.02 - 0.08			
	AIS	0.005 - 0.04			
	AA	0.002 - 0.005			
	PF	0.004 - 0.01			
Kv4.3	Soma	0.002 - 0.004	-88	HH	[10, 31]
Km	AIS	0.0003 - 0.0008	-88	HH	[10, 31]
Kir2.x	Soma	0.0005 - 0.001	-88	HH	[10, 31]
**Ca^{2+} dependent K^+ channels**					
KCa1.1	Dendrites	0.010 - 0.03	-88	Markovian	[35]
**Ca^{2+} channels**					
Cav2.2	Dendrites	0.005 - 0.025	137.5	HH	[10, 31]
	Soma	0.0001 - 0.0007			
	Hillock	0.0001 - 0.0007			
	AIS	0.0001 - 0.0007			
	AA	0.0001 - 0.0007			
	PF				
**TRPM4 like channel**					
TRPM4	Dendrites	$5*10^{-4}$	0	HH	[20]
**Ca^{2+} buffer - Pumps density**					
Ca^{2+} Buffer	Dendrites	$1*10^{-9}$		Markovian	Based on [35] Modified with data from [26, 27]
	Soma	$1*10^{-9}$			
	Hillock	$1*10^{-9}$			
	AIS	$1*10^{-9}$			
	AA	$1*10^{-9}$			
	PF				

The table reports the ionic channels, their location, the range of the maximum conductances and the ionic reversal potential. The corresponding gating equations were written either in Hodgkin-Huxley (HH) style or in Markovian style according to the indicated references.

Supplementary Table 3. Spike features.

	10 pA	***16 pA***	***22 pA***
	Exp	***Exp***	***Exp***
Spike height (mV)	20.93	19.25	18.88
Spike width (mV)	0.70	1.0	1.06
AHP depth (mV)	-71.24	-61.85	-57.25
AHP depth Slow (mV)	-57.93	-52.90	-51.52
Time to first spike (ms)	121.29	107.42	107.10
Mean spike frequency (Hz)	63.54	97.28	98.90
ISI CV	0.18	0.0983	0.088
Spike Count	141	202	207

The table shows exemplar values of features, obtained from experimental traces in a GrC using eFEL.

SUPPLEMENTARY FIGURES

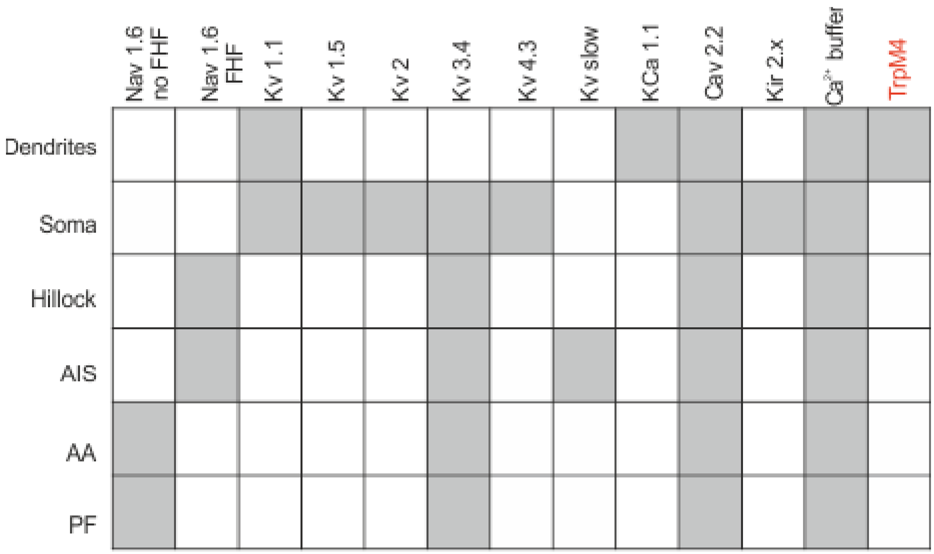

Supplementary Figure 1. Distribution of ionic channels in the GrC model. The table summarizes the ionic channel type and distribution in the different GrC model sections according to literature. The description, for each ionic channel, can be found in the previous section of the Supplemental Material.

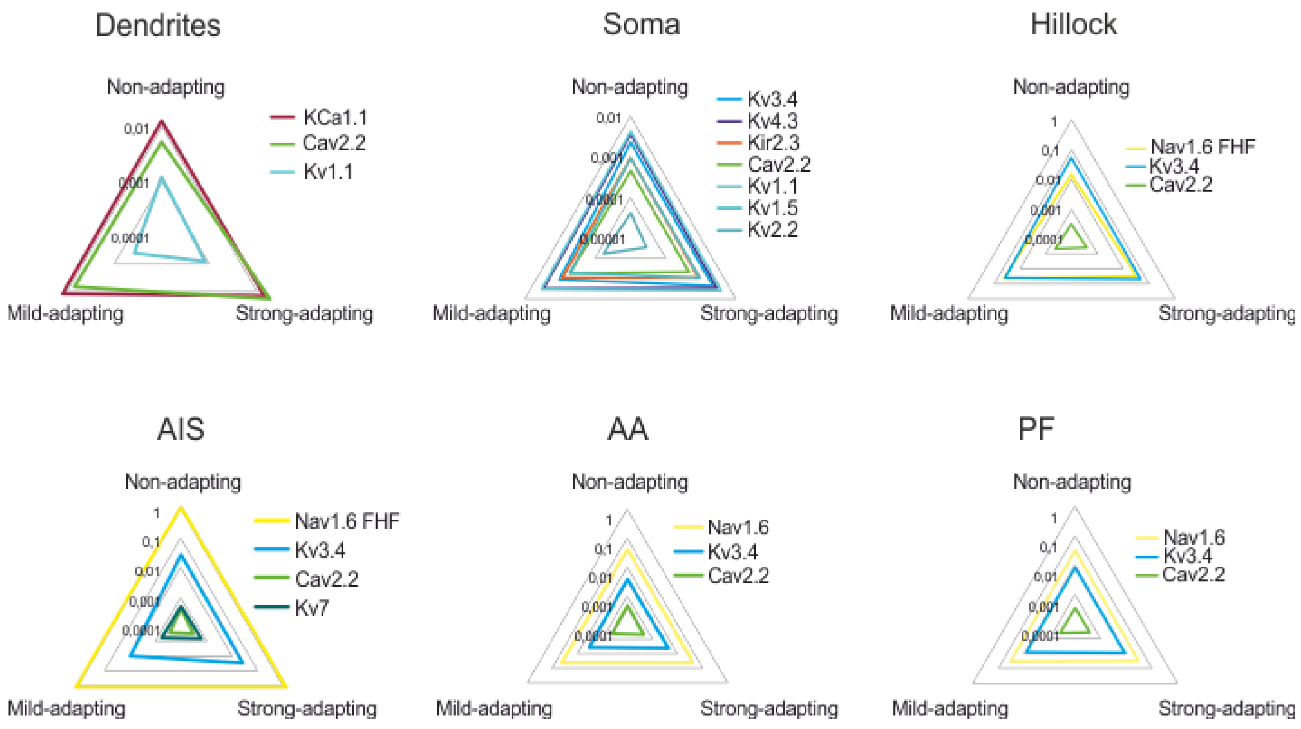

Supplementary Figure 2. Computational modeling of GrC electroresponsiveness. Comparison of average conductance values in the three GrC subtypes. The conductances were obtained from the optimization and describe the differences in each section along the morphology. Notably, strong-adapting GrCs had a markedly higher Cav2.2, slightly higher Nav1.6-FHF and Kv3.4 compared to the other GrCs.

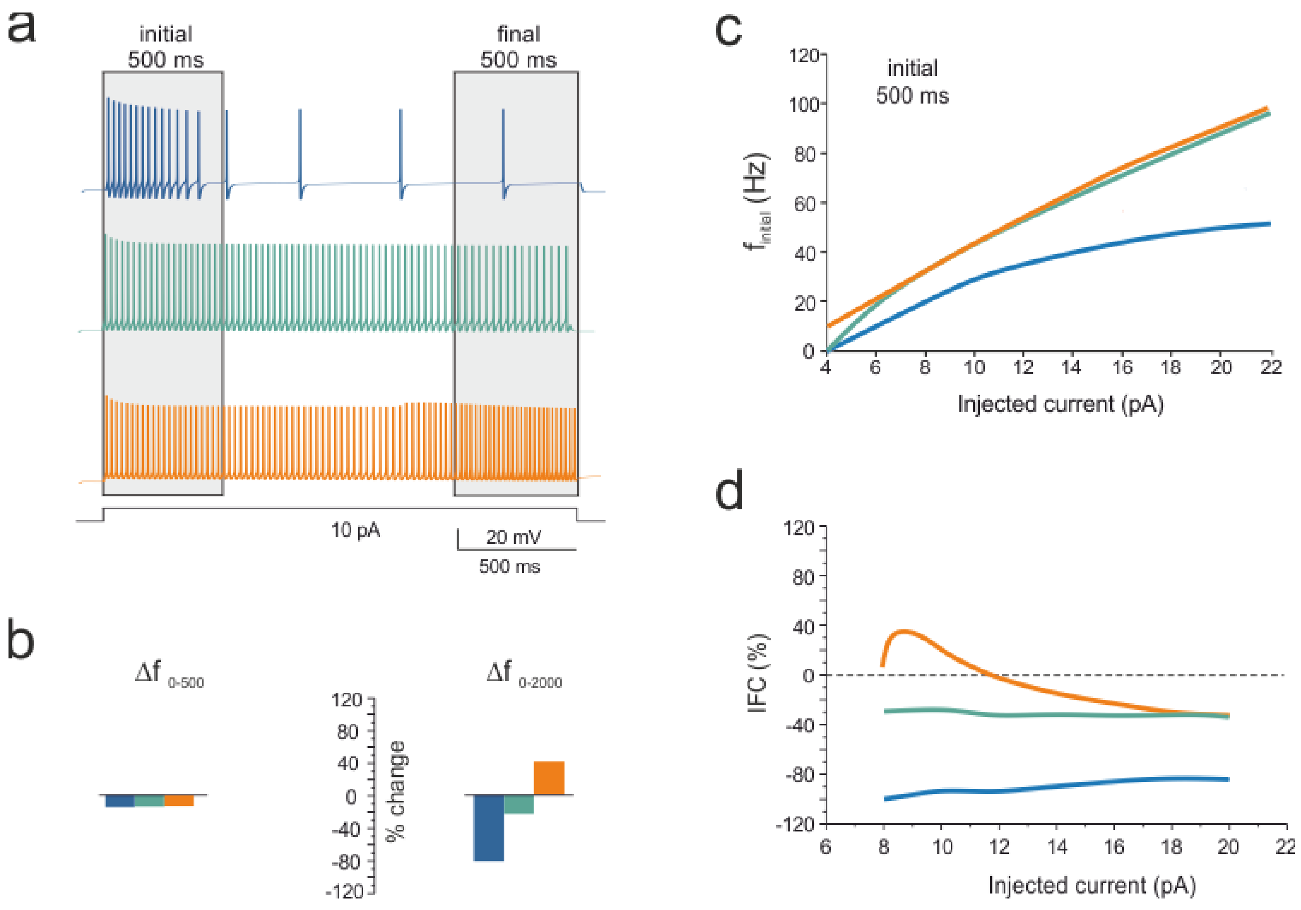

Supplementary Figure 3. Simulation of GrC intrinsic electroresponsiveness. Three exemplar GrC model simulations are shown, one *adapting*, one *non-adapting*, one *accelerating*. The same color codes are used consistently in the figure. (a) Voltage responses to 2000 ms-10 pA current injection from the holding potential of -65 mV. Spike frequency initially remains stable in all the three cells but it shows different trends thereafter. (b) $\Delta f_{0\text{-}500}$ and $\Delta f_{0\text{-}2000}$ are the spike frequency % changes after 500 ms and 2000 ms, respectively. (c) In $f_{initial}/I$ plots, spike frequency increase almost linearly with the injected current intensity in both the accelerating, adapting and non-adapting GrC models. (d) Plot of the intrinsic frequency change IFC *vs.* injected current for the three GrC models. A positive peak is apparent in the accelerating GrC at 10 pA current injection, while negative IFC values prevail in the other GrCs.

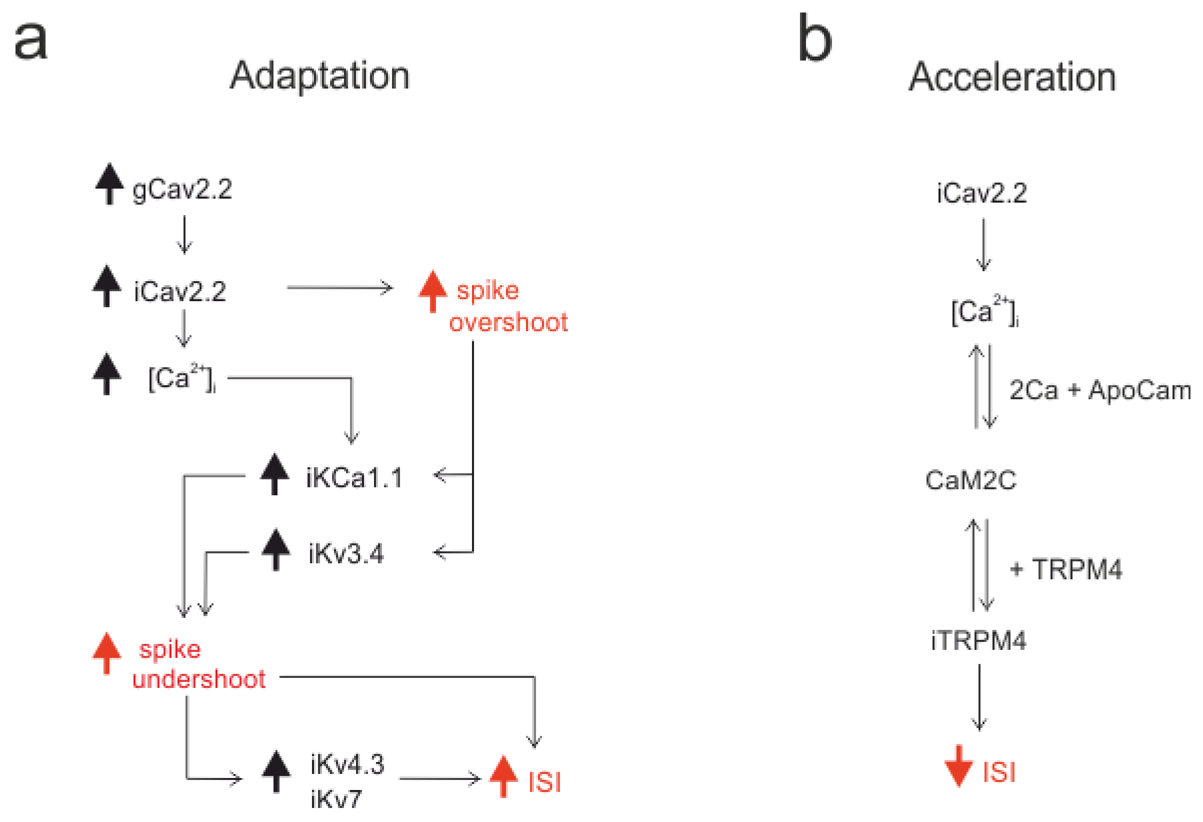

Supplementary Figure 4. Hypothesis on the mechanisms of adaptation and acceleration. (a) *Adaptation.* Schematics of the hypothetic chain of events leading to adaptation. The chain is started by increased Ca^{2+} conductance. (b) *Acceleration.* Schematics of the hypothetic steps required for firing acceleration. The critical step is coupling of Ca^{2+} influx to Calmodulin leading to TRPM4 channel opening.

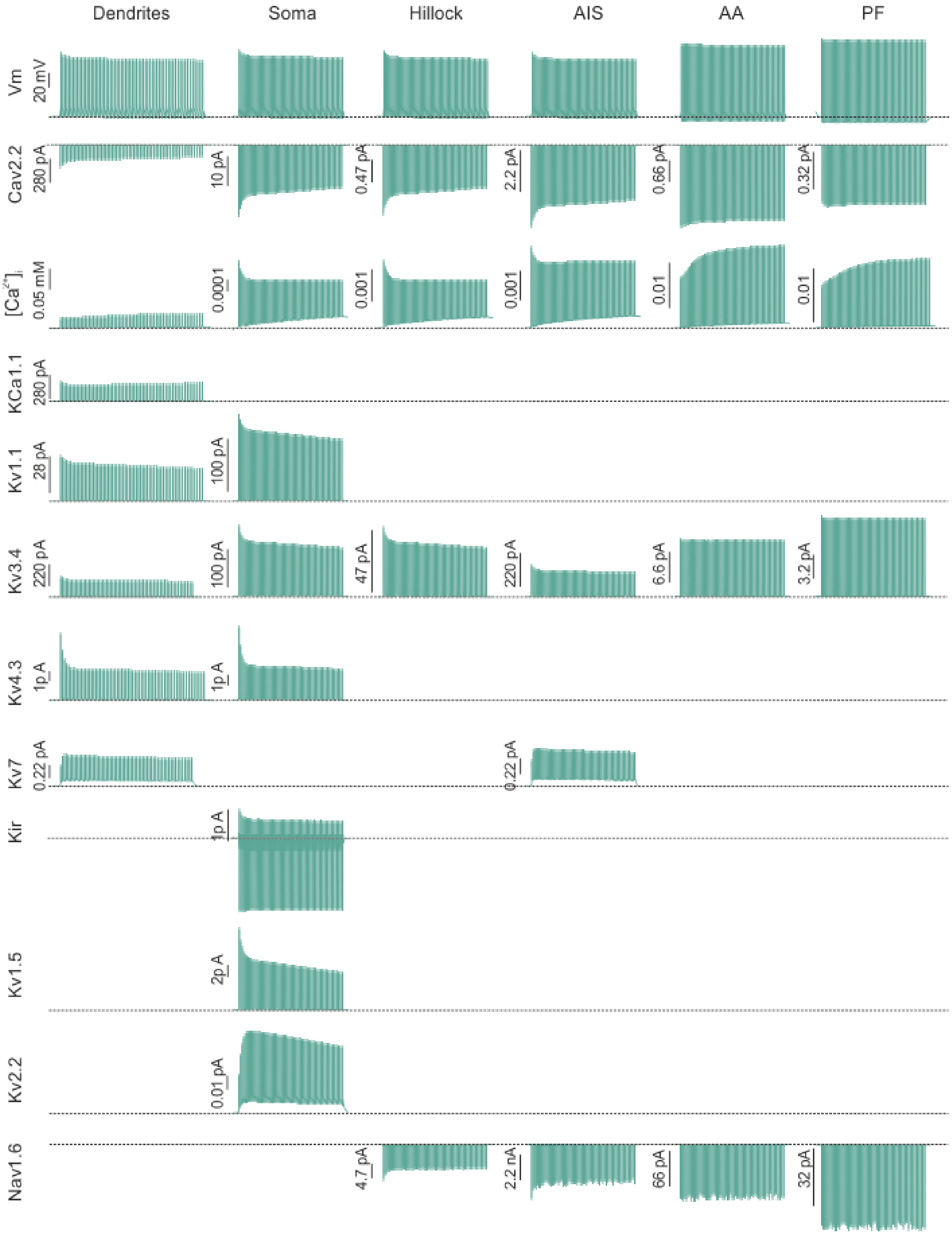

Supplementary Figure 5. Ionic currents in non-adapting GrCs during firing. The currents, for each ionic channel, were recorded from a dendrite, the soma, hillock, AIS, a distal section of AA and a distal section of PF.

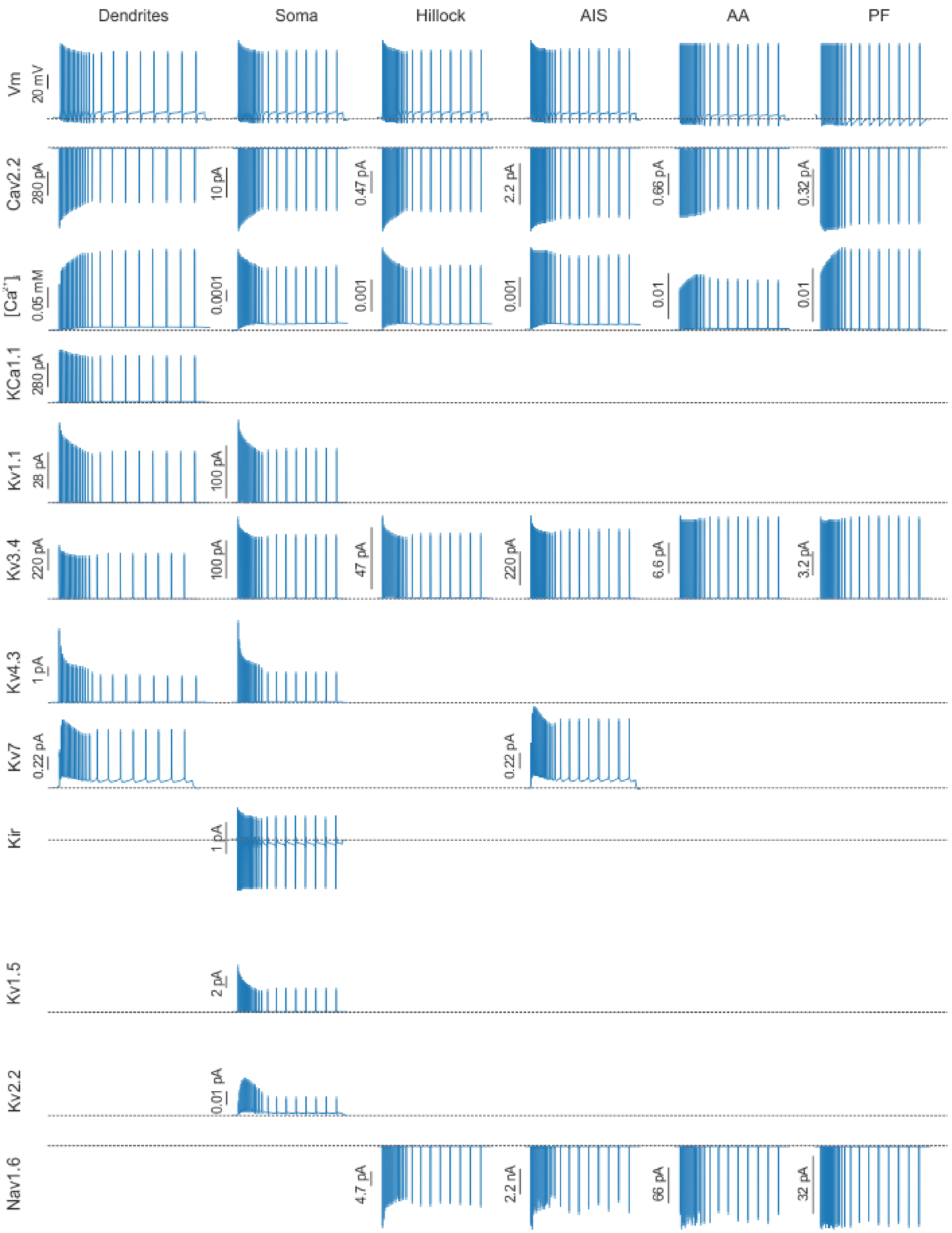

Supplementary Figure 6. Ionic currents in strongly adapting GrCs during firing. The currents, for each ionic channel, were recorded from a dendrite, the soma, hillock, AIS, a distal section of AA and a distal section of PF.

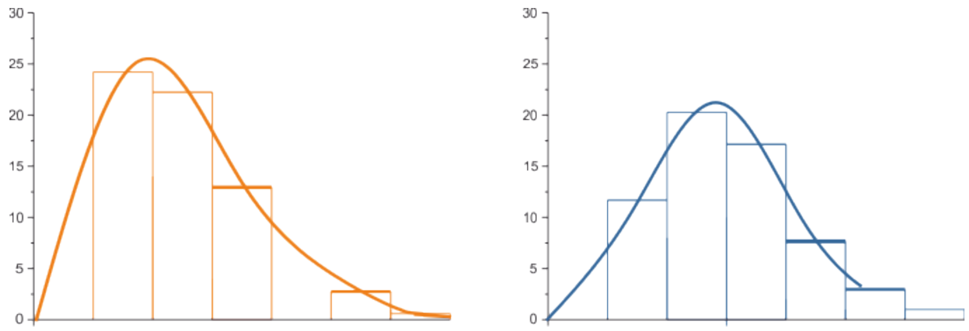

Supplementary Figure 7. The probability density functions of experimental PPRs. Histograms show the experimental PPRs with the corresponding probability density function superimposed through spline interpolations.

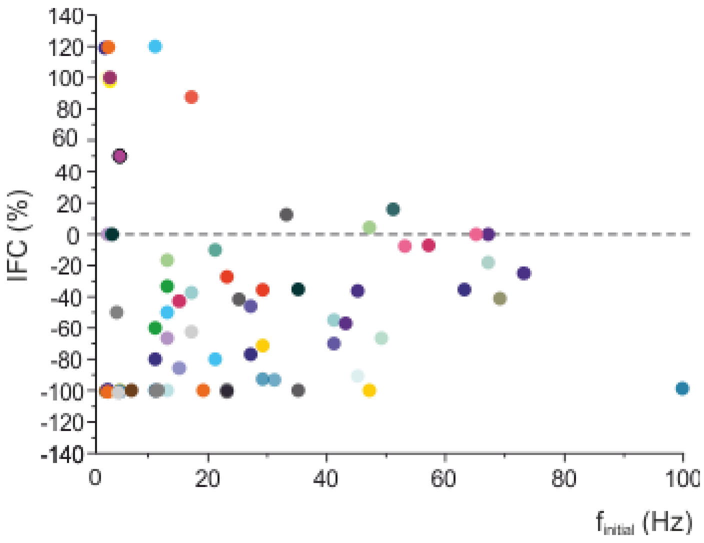

Supplementary Figure 8. Cell properties sorted by experimental sessions. The cell properties used for cluster analysis (cf. Fig. 3a) were sorted by experimental session using colour-codes (a different colour per day). This plot demonstrates that data were interspersed with respect to recording days. It should be noted that the patch-clamp experiments were performed in different days by using different animals and newly prepared solutions. Moreover, slices were changed after each recording. Thus, even in case more GrCs were recorded per day, they belonged to different slices. It is therefore improbable that batch effects might have driven data clustering in Fig. 3a.

SUPPLEMENTARY REFERENCES

1. Khaliq, Z.M., Gouwens, N.W. & Raman, I.M. The contribution of resurgent sodium current to high-frequency firing in Purkinje neurons: an experimental and modeling study. *Journal of Neuroscience* **23**, 4899-4912 (2003).

2. Khaliq, Z.M. & Raman, I.M. Relative contributions of axonal and somatic Na channels to action potential initiation in cerebellar Purkinje neurons. *Journal of Neuroscience* **26**, 1935-1944 (2006).

3. Magistretti, J., Castelli, L., Forti, L. & D'Angelo, E. Kinetic and functional analysis of transient, persistent and resurgent sodium currents in rat cerebellar granule cells in situ: an electrophysiological and modelling study. *The Journal of physiology* **573**, 83-106 (2006).

4. Dover, K., Solinas, S., D'Angelo, E. & Goldfarb, M. Long-term inactivation particle for voltage-gated sodium channels. *The Journal of physiology* **588**, 3695-3711 (2010).

5. Dover, K.*, et al.* FHF-independent conduction of action potentials along the leak-resistant cerebellar granule cell axon. *Nature communications* **7**, 12895-12895 (2016).

6. Goldfarb, M.*, et al.* Fibroblast Growth Factor Homologous Factors Control Neuronal Excitability through Modulation of Voltage-Gated Sodium Channels. *Neuron* **55**, 449-463 (2007).

7. Diwakar, S., Magistretti, J., Goldfarb, M., Naldi, G. & D'Angelo, E. Axonal Na+ channels ensure fast spike activation and back-propagation in cerebellar granule cells. *Journal of neurophysiology* **101**, 519-532 (2009).

8. Chang, S.Y., Zagha, E. & Kwon, E.S. Distribution of Kv3.3 potassium channel subunits in distinct neuronal populations of mouse brain. *Journal of comparative neurology* **972**, 953-972 (2007).

9. Masoli, S., Sergio, S. & Egidio, D.A. Action potential processing in a detailed Purkinje cell model reveals a critical role for axonal compartmentalization. *Frontiers in Cellular Neuroscience* **9**, 1--22 (2015).

10. D'Angelo, E., *et al.* Theta-frequency bursting and resonance in cerebellar granule cells: experimental evidence and modeling of a slow k+-dependent mechanism. *The Journal of neuroscience : the official journal of the Society for Neuroscience* **21**, 759-770 (2001).

11. Khavandgar, S., Walter, J.T., Sageser, K. & Khodakhah, K. Kv1 channels selectively prevent dendritic hyperexcitability in rat Purkinje cells. *The Journal of physiology* **569**, 545-557 (2005).

12. Chung, Y.H., Shin, C., Kim, M.J., Lee, B.K. & Cha, C.I. Immunohistochemical study on the distribution of six members of the Kv1 channel subunits in the rat cerebellum. *Brain Res* **895**, 173-177 (2001).

13. Debanne, D., Campanac, E., Bialowas, A., Carlier, E. & Alcaraz, G. Axon physiology. *Physiol Rev* **91**, 555-602 (2011).

14. Ranjan, R., *et al.* Channelpedia: An Integrative and Interactive Database for Ion Channels. *Frontiers in Neuroinformatics* **5**, 1-8 (2011).

15. Cooper, E.C. Made for “anchorin”: Kv7.2/7.3 (KCNQ2/KCNQ3) channels and the modulation of neuronal excitability in vertebrate axons. *Seminars in Cell & Developmental Biology* **22**, 185-192 (2011).

16. Rossi, P., D'Angelo, E., Magistretti, J., Toselli, M. & Taglietti, V. Age-dependent expression of high-voltage activated calcium currents during cerebellar granule cell development in situ. *Pflugers Arch* **429**, 107-116 (1994).

17. D'Angelo, E., De Filippi, G., Rossi, P. & Taglietti, V. Synaptic activation of Ca2+ action potentials in immature rat cerebellar granule cells in situ. *J Neurophysiol* **78**, 1631-1642 (1997).

18. Rossi, P., *et al.* Inhibition of constitutive inward rectifier currents in cerebellar granule cells by pharmacological and synaptic activation of GABAB receptors. *European Journal of Neuroscience* **24**, 419-432 (2006).

19. Rossi, P., De Filippi, G., Armano, S., Taglietti, V. & D'Angelo, E. The weaver mutation causes a loss of inward rectifier current regulation in premigratory granule cells of the mouse

cerebellum. *J Neurosci* **18**, 3537-3547 (1998).

20. Subramaniyam, S.*, et al.* Computational modeling predicts the ionic mechanism of late-onset responses in unipolar brush cells. *Front Cell Neurosci* **8**, 237 (2014).

21. Pepke, S., Kinzer-Ursem, T., Mihalas, S. & Kennedy, M.B. A dynamic model of interactions of Ca2+, calmodulin, and catalytic subunits of Ca2+/calmodulin-dependent protein kinase II. *PLoS Comput Biol* **6**, e1000675 (2010).

22. Nilius, B.*, et al.* Regulation of the Ca2+ sensitivity of the nonselective cation channel TRPM4. *J Biol Chem* **280**, 6423-6433 (2005).

23. Gall, D.*, et al.* Altered neuronal excitability in cerebellar granule cells of mice lacking calretinin. *The Journal of neuroscience : the official journal of the Society for Neuroscience* **23**, 9320-9327 (2003).

24. Schwaller, B. Calretinin: from a "simple" Ca(2+) buffer to a multifunctional protein implicated in many biological processes. *Frontiers in neuroanatomy* **8**, 3-3 (2014).

25. Masoli, S.*, et al.* Single Neuron Optimization as a Basis for Accurate Biophysical Modeling: The Case of Cerebellar Granule Cells. *Frontiers in Cellular Neuroscience* **11**, 1-14 (2017).

26. Faas, G.C., Schwaller, B., Vergara, J.L. & Mody, I. Resolving the fast kinetics of cooperative binding: Ca2+ buffering by calretinin. *PLoS Biology* **5**, 2646-2660 (2007).

27. Saftenku, E.È. Effects of Calretinin on Ca2+ Signals in Cerebellar Granule Cells: Implications of Cooperative Ca2+ Binding. *The Cerebellum* **11**, 102-120 (2012).

28. Bender, K.J. & Trussell, L.O. The Physiology of the Axon Initial Segment. *Annual Review of Neuroscience* **35**, 249-265 (2012).

29. Wilms, C.D. & Häusser, M. Reading out a spatiotemporal population code by imaging neighbouring parallel fibre axons in vivo. *Nature Communications* **6**, 6464-6464 (2015).

30. D'Angelo, E., De Filippi, G., Rossi, P. & Taglietti, V. Synaptic excitation of individual rat cerebellar granule cells in situ: evidence for the role of NMDA receptors. *The Journal of physiology* **484 (Pt 2**, 397-413 (1995).

31. Diwakar, S., Magistretti, J., Goldfarb, M., Naldi, G. & D'Angelo, E. Axonal Na+ channels ensure fast spike activation and back-propagation in cerebellar granule cells. *Journal of neurophysiology* **101**, 519-532 (2009).

32. Akemann, W. & Knopfel, T. Interaction of Kv3 Potassium Channels and Resurgent Sodium Current Influences the Rate of Spontaneous Firing of Purkinje Neurons. *Channels* **26**, 4602-4612 (2006).

33. Courtemanche, M., Ramirez, R.J. & Nattel, S. Ionic mechanisms underlying human atrial action potential properties: insights from a mathematical model. *The American journal of physiology* **275**, H301-321 (1998).

34. Raman, I.M. & Bean, B.P. Inactivation and recovery of sodium currents in cerebellar Purkinje neurons: evidence for two mechanisms. *Biophysical Journal* **80**, 729-737 (2001).

35. Anwar, H., Hong, S. & De Schutter, E. Controlling Ca2+-Activated K+ Channels with Models of Ca2+ Buffering in Purkinje Cells. *The Cerebellum*, 1-13 (2010).
