# Supplementary material for: Parameter tuning differentiates granule cell subtypes enriching transmission properties at the cerebellum input stage
Source: Commun Biol. 2020 May 8;3:222. doi: 10.1038/s42003-020-0953-x (PMC7210112; doi:10.1038/s42003-020-0953-x)
Supplement: Supplementary file 3 — Description of Additional Supplementary Files [file 42003_2020_953_MOESM3_ESM.pdf]

Description of Additional Supplementary Files

File Name: **Supplementary Data**

Description: The source data corresponding to graphs and charts presented in the main figures are reported. The Supplementary Data file is organized into sheets containing the data of graphs and figures.
