# Supplementary material for: Parameter tuning differentiates granule cell subtypes enriching transmission properties at the cerebellum input stage
Source: Commun Biol. 2020 May 8;3:222. doi: 10.1038/s42003-020-0953-x (PMC7210112; doi:10.1038/s42003-020-0953-x)
Supplement: Supplementary file 4 — Reporting Summary [file 42003_2020_953_MOESM4_ESM.pdf]

nature research

Corresponding author(s): Egidio D'Angelo

Last updated by author(s): Mar 24, 2020

Reporting Summary

Nature Research wishes to improve the reproducibility of the work that we publish. This form provides structure for consistency and transparency in reporting. For further information on Nature Research policies, see Authors & Referees and the Editorial Policy Checklist.

Statistics

For all statistical analyses, confirm that the following items are present in the figure legend, table legend, main text, or Methods section.

n/a	Confirmed	
☐	☒	The exact sample size (*n*) for each experimental group/condition, given as a discrete number and unit of measurement
☒	☐	A statement on whether measurements were taken from distinct samples or whether the same sample was measured repeatedly
☒	☐	The statistical test(s) used AND whether they are one- or two-sided<br>*Only common tests should be described solely by name; describe more complex techniques in the Methods section.*
☒	☐	A description of all covariates tested
☒	☐	A description of any assumptions or corrections, such as tests of normality and adjustment for multiple comparisons
☐	☒	A full description of the statistical parameters including central tendency (e.g. means) or other basic estimates (e.g. regression coefficient) AND variation (e.g. standard deviation) or associated estimates of uncertainty (e.g. confidence intervals)
☐	☒	For null hypothesis testing, the test statistic (e.g. *F*, *t*, *r*) with confidence intervals, effect sizes, degrees of freedom and *P* value noted<br>*Give P values as exact values whenever suitable.*
☒	☐	For Bayesian analysis, information on the choice of priors and Markov chain Monte Carlo settings
☒	☐	For hierarchical and complex designs, identification of the appropriate level for tests and full reporting of outcomes
☒	☐	Estimates of effect sizes (e.g. Cohen's *d*, Pearson's *r*), indicating how they were calculated

Our web collection on statistics for biologists contains articles on many of the points above.

Software and code

Policy information about availability of computer code

Data collection	pClamp10 (Clampex10.4), Multiclamp700B, eFEL
Data analysis	pClamp10 (Clampfit10), MS Excel 2007, Matlab 2015, OriginPro8, NEURON, BluePyOpt

For manuscripts utilizing custom algorithms or software that are central to the research but not yet described in published literature, software must be made available to editors/reviewers. We strongly encourage code deposition in a community repository (e.g. GitHub). See the Nature Research guidelines for submitting code & software for further information.

Data

Policy information about availability of data

All manuscripts must include a data availability statement. This statement should provide the following information, where applicable:

- Accession codes, unique identifiers, or web links for publicly available datasets
- A list of figures that have associated raw data
- A description of any restrictions on data availability

The data can be requested to the Authors and are available on the Knowledge Graph (Human Brain Project, https://kg.ebrains.eu/search/live/minds/core/dataset/v1.0.0/7dc5d5d5-4323-41d6-bdfd-0b841cfe7000);
The code is available on the Brain Simulation Platform (Human Brain Project), where models are showcased as a “live paper” on Python notebooks for optimization and simulation (https://humanbrainproject.github.io/hbp-bsp-live-papers/2019/masoli_et_al_2019/masoli_et_al_2019.html).

Field-specific reporting

Please select the one below that is the best fit for your research. If you are not sure, read the appropriate sections before making your selection.

☒ Life sciences ☐ Behavioural & social sciences ☐ Ecological, evolutionary & environmental sciences

For a reference copy of the document with all sections, see nature.com/documents/nr-reporting-summary-flat.pdf

Life sciences study design

All studies must disclose on these points even when the disclosure is negative.

Sample size	The sample size was determined on the basis of literature and in order to guarantee a significance level.
Data exclusions	An experimental trace was discarded when the cell series resistance did not remain stable during recordings;<br>a model was discarded when (1) spike generation in the axonal initial segment failed, (2) spike conduction speed or spike amplitude in the ascending axon and parallel fibers was decremental, (3) spike frequency was different in soma and axon (>± 1 spike/sec).
Replication	All attempts at replication were successful.
Randomization	no randomization needed
Blinding	no blinding needed

Reporting for specific materials, systems and methods

We require information from authors about some types of materials, experimental systems and methods used in many studies. Here, indicate whether each material, system or method listed is relevant to your study. If you are not sure if a list item applies to your research, read the appropriate section before selecting a response.

Materials & experimental systems

n/a	Involved in the study	
☐	☒	Antibodies
☒	☐	Eukaryotic cell lines
☒	☐	Palaeontology
☐	☒	Animals and other organisms
☒	☐	Human research participants
☒	☐	Clinical data

Methods

n/a	Involved in the study	
☒	☐	ChIP-seq
☒	☐	Flow cytometry
☒	☐	MRI-based neuroimaging

Antibodies

Antibodies used	Rabbit polyclonal anti-TRPM4 (Cat.# ACC-044; Alomone labs, Jerusalem, Israel); rabbit recombinant Anti-PAX6 antibody [EPR15858] (ab195045; Abcam, Cambridge, UK). Secondary antibodies: anti-rabbit IgG F(ab')2 fragment (Alexa Fluor® 488) conjugated (#4412; Cell Signaling technology Inc.) diluted 1:350 in blocking solution and Donkey Fab Rabbit IgG (H&L) Antibody Rhodamine Conjugated (# 811-7002; Rockland Immunochemicals, Inc., USA) diluted 1:1000 in blocking solution.
Validation	WB, KO

Animals and other organisms

Policy information about studies involving animals; ARRIVE guidelines recommended for reporting animal research

Laboratory animals	Wistar rats, either sex, from 18 to 24 days old.
Wild animals	The study did not involve wild animals.
Field-collected samples	The study did not involve samples collected from the field.
Ethics oversight	Ethical committee of Italian Ministry of Health.

Note that full information on the approval of the study protocol must also be provided in the manuscript.
